# Longitudinal RNA Sequencing of Skin and DRG Neurons in Mice with Paclitaxel-Induced Peripheral Neuropathy

**DOI:** 10.3390/data7060072

**Published:** 2022-05-30

**Authors:** Anthony M. Cirrincione, Cassandra A. Reimonn, Benjamin J. Harrison, Sandra Rieger

**Affiliations:** 1 Department of Biology, University of Miami, Coral Gables, FL 33146, USA; 2 Department of Biomedical Sciences, University of New England, Biddeford, ME 04005, USA; 3 Sylvester Comprehensive Cancer Center, Miller School of Medicine, University of Miami, Miami, FL 33136, USA

**Keywords:** paclitaxel, CIPN, peripheral neuropathy, RNAseq, chemotherapy, skin, dorsal root ganglion neurons, pain, paclitaxel-induced peripheral neuropathy, Taxol

## Abstract

Paclitaxel-induced peripheral neuropathy is a condition of nerve degeneration induced by chemotherapy, which afflicts up to 70% of treated patients. Therapeutic interventions are unavailable due to an incomplete understanding of the underlying mechanisms. We previously discovered that major physiological changes in the skin underlie paclitaxel-induced peripheral neuropathy in zebrafish and rodents. The precise molecular mechanisms are only incompletely understood. For instance, paclitaxel induces the upregulation of MMP-13, which, when inhibited, prevents axon degeneration. To better understand other gene regulatory changes induced by paclitaxel, we induced peripheral neuropathy in mice following intraperitoneal injection either with vehicle or paclitaxel every other day four times total. Skin and dorsal root ganglion neurons were collected based on distinct behavioural responses categorised as “pain onset” (d4), “maximal pain” (d7), “beginning of pain resolution” (d11), and “recovery phase” (d23) for comparative longitudinal RNA sequencing. The generated datasets validate previous discoveries and reveal additional gene expression changes that warrant further validation with the goal to aid in the development of drugs that prevent or reverse paclitaxel-induced peripheral neuropathy.

## 1. Summary

Paclitaxel-induced peripheral neuropathy is a condition of nerve degeneration induced by chemotherapy, which afflicts up to 70% of treated patients. Therapeutic interventions are unavailable due to an incomplete understanding of the underlying mechanisms. We previously discovered that major physiological changes in the skin underlie paclitaxel-induced peripheral neuropathy in zebrafish and rodents. The precise molecular mechanisms are only incompletely understood. For instance, paclitaxel induces the upregulation of MMP13, which, when inhibited, prevents axon degeneration. To better understand other gene regulatory changes induced by paclitaxel, we induced peripheral neuropathy in mice following intraperitoneal injection either with vehicle or paclitaxel every other day four times total. Skin and dorsal root ganglion neurons were collected based on distinct behavioural responses categorised as “pain onset” (d4), “maximal pain” (d7), “beginning of pain resolution” (d11), and “recovery phase” (d23) for comparative longitudinal RNA sequencing. The generated datasets validate previous discoveries and reveal additional gene expression changes that warrant further validation with the goal to aid in development of drugs that prevent or reverse paclitaxel-induced peripheral neuropathy.

## 2. Data Description

### 2.1. Background and Summary

Peripheral neuropathy is a common side effect of chemotherapy characterised by paraesthesia (tingling), numbness, pain, temperature sensitivity, and motor weakness. Paclitaxel (Taxol) is one of the most widely used chemotherapeutic agents, which primarily affects the somatosensory neurons innervating the skin [1–3]. Pathological examinations suggest that intraepidermal unmyelinated axons are the first to degenerate upon paclitaxel treatment [4–8]. Thus, understanding the genetic mechanisms underlying the earliest manifestations of the disease will be essential to develop therapies that allow chemotherapy patients to complete cancer treatment without disruption, and prevent irreversible long-term symptoms.

Few studies have established expression profiles of chemotherapy-induced peripheral neuropathy. In one study, parallel gene expression profiles from dorsal root ganglion (DRG) neurons in mice were established following injection with the chemotherapeutic agents, oxaliplatin, vincristine, and cisplatin [9]. This comparative study revealed that only few genes were common among these data sets, suggesting that fundamental differences in the aetiology of chemotherapy-induced peripheral neuropathy (CIPN) must exist. This may not be surprising given the differences in the mechanisms of action for each of these chemotherapeutic agents, leading to potentially different off-target effects. In addition, the investigation of dorsal root ganglion (DRG) neurons may have obscured common upstream mechanisms. For instance, we previously showed that sensory neurons are secondarily affected by earlier epidermal damage, which promotes the degeneration of intraepidermal nerve endings in zebrafish, rats, and mice treated with paclitaxel. Epidermal keratinocytes are damaged due to increased reactive oxygen species formation and upregulation of matrix metalloproteinases, such as MMP-13, leading to extracellular matrix damage that ultimately affects the axons, leading to their degeneration [8]. Epidermal damage can be prevented when animals are treated with pharmacological MMP-13 inhibitors [10]. Therefore, the skin plays a crucial role in sensory axon homeostasis.

Existing genomic studies have focused on single time point analyses and single-cell types [11,12]. For instance, RNA sequencing was used to analyse blood samples of breast cancer survivors who suffered from long-term paclitaxel-induced peripheral neuropathy and these samples were compared to breast cancer survivors without neuropathy [13]. This study identified changes in mitochondrial genes that had been previously identified in preclinical CIPN models as differentially regulated, validating the importance of these models in studying human pathology [13]. Potentially, mitochondrial dysfunction might play a role in the deficiency of some patients to resolve their neuropathy symptoms. Despite these findings, longitudinal studies to detect changes in affected tissues over prolonged time periods have not been conducted, and currently, no data is available on gene expression changes prior to the onset of neuropathic symptoms. This information, however, will be critical to understanding the molecular gene expression networks involved in the onset, progression, and resolution of neuropathy.

**Figure 1.**
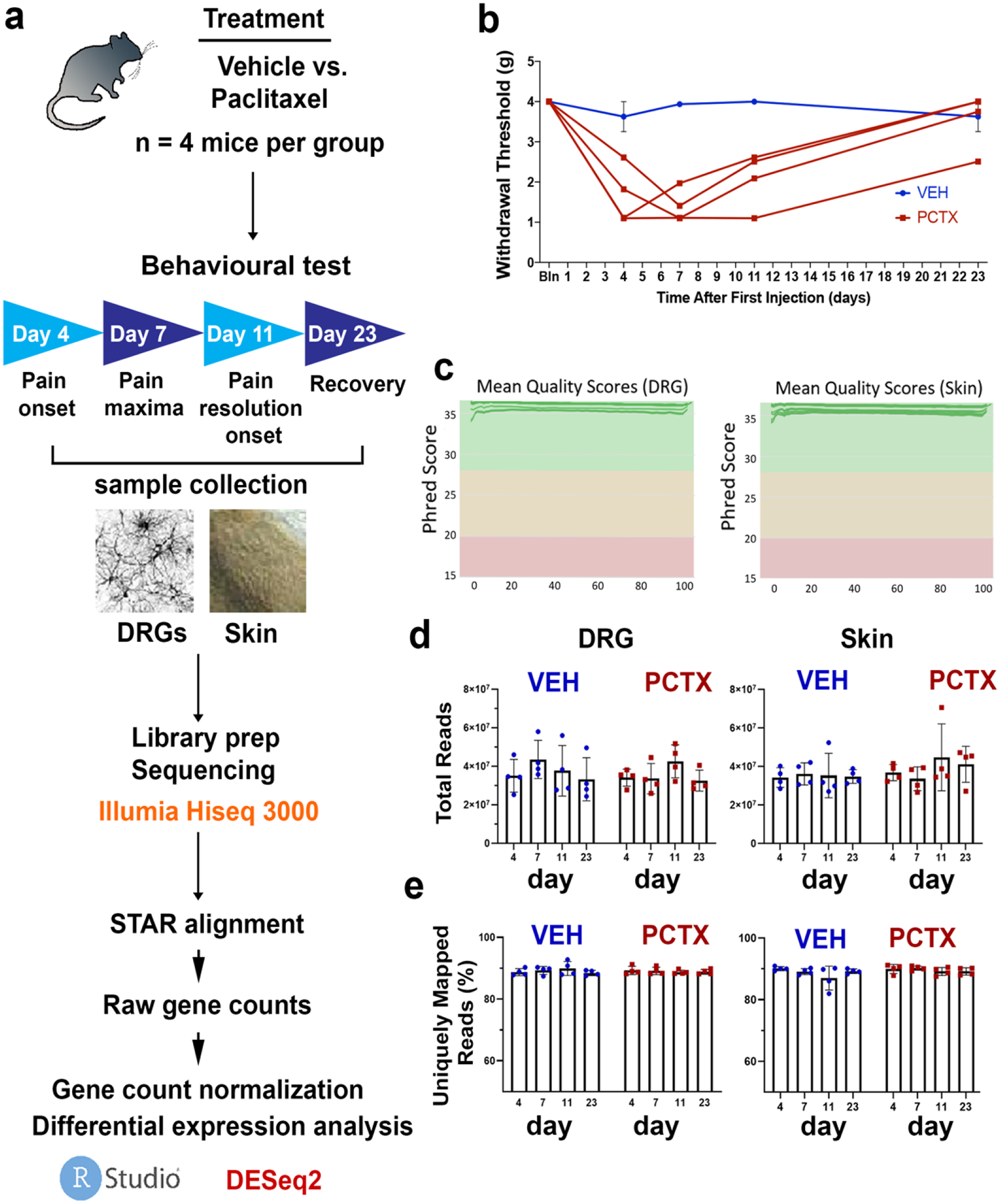
Experimental overview of longitudinal RNAseq analysis comparing dorsal root ganglion neurons and skin from paclitaxel- and vehicle-treated mice. (**a**) Summary of experimental workflow from treatment and sample collection to data trimming and analysis. (**b**) Behavioural analysis using von Frey filaments to assess the touch response showing the development of paclitaxel-induced neuropathy in mice evident by reduced pain threshold at 4, 7, and 11 days with a subsequent recovery (*n* = 4 per treatment group per time point). (**c**) Read-quality scores for DRG neurons (**left**) and skin (right). (**d**,**e**) Total and uniquely mapped reads among DRG (left) and skin (**right**) samples from paclitaxel- and vehicle-treated animals. *Abbreviations*: VEH: vehicle; PCTX: Paclitaxel.

To address this need, we performed a comprehensive RNAseq study using skin and DRG neuron samples of vehicle and paclitaxel-treated mice. We compared gene expression profiles according to pain profiles generated in these mice. Mice were injected four times every other day with either vehicle or paclitaxel and subsequently underwent a recovery period between Day 7 and Day 23. Tissues were collected and analysed during these time points, which were categorised as “pain onset” on Day 4, “maximal pain sensitivity” on Day 7, “beginning of pain resolution” on Day 11, and “post pain” on Day 23 (Figure 1). The generated data sets will be useful for the research community to further validate the genes implicated in paclitaxel-induced peripheral neuropathy.

### 2.2. Technical Validation

#### 2.2.1. Differential Expression Analysis

Gene counts from STAR alignment were imported into the R environment using Rstudio (R Version 4.0.3; R studio version 1.3.1093; Peter Dalgaard; Frederiksberg, Denmark) and the package *base* version 4.0.3. The package *dplyr* version 1.0.5 was used to construct individual raw gene count matrices per tissue type, skin, and dorsal root ganglion neurons (DRGs), each consisting of 32 samples combined from both treatment groups. Normalised gene expression for both tissue data sets was calculated using the default parameters in the Deseq2 package version 1.3.1 (Michael Love; Chapel Hill, NC, USA).

#### 2.2.2. Expression of Housekeeping and Validation Genes

**Figure 2.**
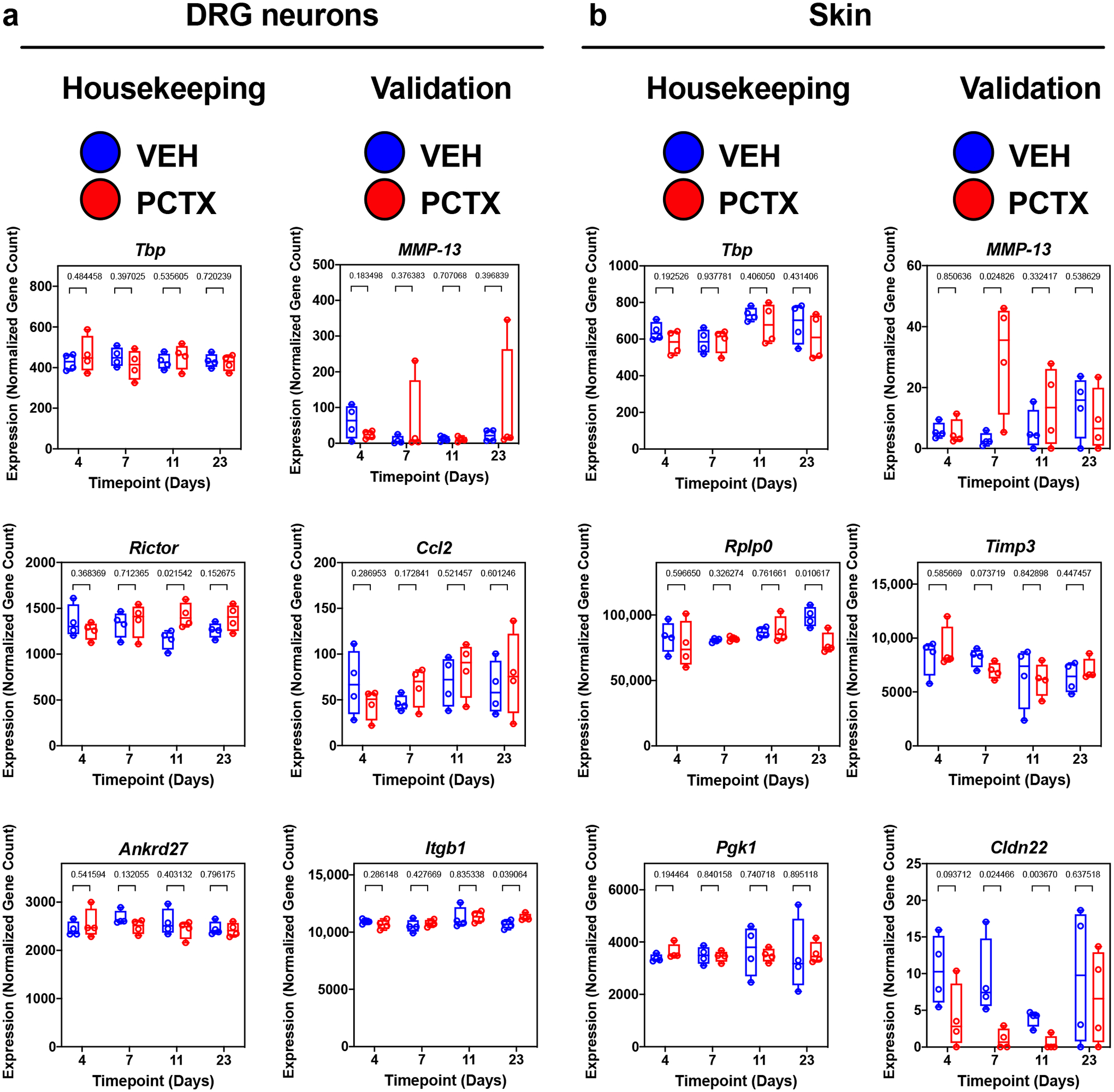
Technical validation of genes. Expression graphs of normalised gene counts showing housekeeping genes (no expected expression changes) and validation genes (expected expression changes) for (**a**) DRG neurons and (**b**) skin. (**a**) DRG validation genes include *Ccl2* and *Itgb1*, known to be overexpressed in DRG neurons in the presence of paclitaxel, and *Mmp13*, only known to play a skin-specific role in paclitaxel-induced peripheral neuropathy. (**b**) Known housekeeping genes in skin include *Tbp, RplpO*, and *Pgk1*. Validation genes in skin include *Mmp13*, *Timp3*, a known inhibitor of MMP-13, and *Cldn22*, a tight junction protein in the epidermis. *p*-values from a two-sided *t*-test are represented above brackets for each time point. *Abbreviations*: VEH: vehicle; PCTX: Paclitaxel.

Normalised gene counts were plotted using Prism 9 (GraphPad Prism Version 9.0.0; San Diego, CA, USA). Previously validated housekeeping genes for DRG and skin were plotted in addition to genes shown to be overexpressed during paclitaxel-induced peripheral neuropathy in either one. To validate DRG-specific housekeeping genes, we plotted *TATA box binding protein* (*Tbp*)*, RPTOR independent companion of MTOR, complex 2* (*Rictor*), and *Ankyrin repeat domain-containing protein 27* (*Ankrd27*), as these are known to be unaffected in their expression levels during nerve injury and pain [14] (Figure 2a). Next, we plotted genes that have been established to be overexpressed in DRG neurons in the presence of paclitaxel, including *Monocyte chemoattractant protein 1* (*Ccl2*). This gene mediates macrophage recruitment and promotes peripheral neuropathy in the presence of paclitaxel [15]. We further analysed the expression of *Itgb1*, which protects DRG neurons from paclitaxel-induced axon damage [16]. This revealed its upregulation, potentially a compensatory mechanism due to altered *Itgb1* trafficking in DRG neurons of paclitaxel-treated mice [16]. Since our research showed that increased MMP-13 activity in the epidermis promotes the development of paclitaxel-induced peripheral neuropathy, we further determined whether Mmp13 expression also changes in DRG neurons, which revealed no significant increase in these neurons.

To validate skin-specific gene expression profiles, we choose to analyse the known keratinocyte housekeeping genes, *TATA box binding protein* (*Tbp)*, *Ribosomal protein large P0* (*Rplp0*), and *Phosphoglycerate kinase 1* (*Pgk1*) [17,18], which as expected did not significantly vary in their expression levels regardless of treatment (Figure 2b). To validate genes that we expected to display a change in expression, we first analysed *Mmp13*, which displayed a paclitaxel-dependent significant increase in expression in the skin, in line with our previous findings [2,8]. Next, we analysed the expression of *Tissue inhibitor of metalloproteinase-3* (*Timp3*), a known antagonist of MMP-13 [19], which displayed a downward regulation trend; however, this was not significant. Given the altered cell adhesion in the skin of paclitaxel-treated animals induced by increased MMP activity, we also analysed the tight junction protein, *Claudin 22* (*Cldn22*) [2,8]. Tight junctions, for instance, have been shown to be decreased upon MMP-13 activation in intestinal epithelia upon LPS stimulation [20]. Consistent with this finding, we detected a significant decrease in *Cldn22* expression in the skin [21] following paclitaxel treatment. This confirms that expected gene expression trends can be detected in our gene expression data sets, thus validating our approach. These data will be useful for the research community for further analysis. Table 1 provides a description for each of the individual raw files, their corresponding sample ID, and information about the time points, treatment, and tissue type.

## 3. Methods

### 3.1. Animals

All animal procedures were approved by the University of New England Institutional Animal Care and Use Committee. Adult male C57BL6/J mice (JAX) weighing 20–25 g were purchased from the Jackson Laboratory. Upon arrival, mice were housed 4/cage and allowed to acclimate to the facility for 7 days. All animals were kept on a 12 h light/dark cycle with ad libitum access to food and water.

### 3.2. Paclitaxel Treatment

The experimental design, time points, and downstream analyses are depicted in Figure 1a. Paclitaxel was administered on Days 0, 2, 4, and 6. Paclitaxel (Sigma-Aldrich, St. Louis, USA) was dissolved in (1:1 Cremophor: Ethanol) and further diluted in 0.9% NaCl to make a final concentration of 0.4 mg/mL. Six-week-old mice were injected intraperitoneally with either vehicle or paclitaxel at a volume of 10 mL/kg bodyweight to make a final concentration of 4 mg/kg (cumulative 16 mg/kg).

### 3.3. Analysis of Tactile Thresholds

Tactile allodynia was quantified (in the mornings prior to injections when assessed on injection days) by measuring the hind paw withdrawal threshold to von Frey filament stimulation, using the up–down method as previously reported [22]. Results are shown in Figure 1b. Animals were placed in a clear Plexiglas chamber and allowed to habituate for ~60 min. Touch-Test filaments (North Coast Medical, Morgan Hill, CA, USA) were used for all testing. The filament range was: 2.44, 2.83, 3.22, 3.61, 4.08, 4.31, 4.56, starting with 3.61. Withdrawal thresholds were determined by sequentially increasing and decreasing the stimulus intensity (“up and down” method). This up–down method was stopped four measures after the first positive response. The response threshold was subsequently analysed by using a Dixon nonparametric test and expressed as the paw withdrawal threshold in gram force values [23]. A clear paw withdrawal, shaking, or licking was considered as a positive or painful response.

### 3.4. Tissue Collection

Animals were exsanguinated by intracardial perfusion of 100 mL ice-cold heparinised phosphate-buffered saline (PBS). Tissues were then immediately harvested on ice before flash freezing and stored at −80 °C. DRG neurons were harvested from left lumbar segments L3, L4, and L5. Plantar skin was taken from the left hind paw. Tissues were harvested at time points consistent with the development of paclitaxel-induced neuropathy in rodents characterised as “pain onset” on Day 4, “maximal pain sensitivity” on Day 7, “beginning of pain resolution” on Day 11, and “post pain” on Day 23 [10].

### 3.5. RNA Extraction and Quality Control

DRG were homogenised using a glass-Teflon homogeniser on ice for 2 min with 350 μL buffer RLT plus 2-Mercaptoethanol (Qiagen, Boston, MA, USA). RNA was extracted using Qiagen RNeasy microcolumns according to the manufacturer’s protocol and eluted with 30 μL ultrapure RNase-free water. Skin was homogenised with a rotor–stator homogeniser for 2 min on ice with TRIzol reagent (Thermo, St. Louis, USA) before phase separation with phase lock gel microcentrifuge tubes (Eppendorf, Framington, MA, USA) to prevent guanidine salt contamination, according to the manufacturer’s protocol. RNA purity was confirmed using a nanodrop UV spectrometer (260/280 ratio > 2), and RNA quality was assessed with the Agilent 2100 Bioanalyzer (RIN > 7).

### 3.6. cDNA Library Preparation and Sequencing

Ribosomal RNA-depleted cDNA libraries were prepared using KAPA RNA HyperPrep kits with RiboErase (KAPA Biosystems) according to the manufacturer’s protocol. Libraries were sequenced with an Illumina Hi-seq 3000 (single-end, 1 × 100 bp).

### 3.7. Sequence Alignment, Counts, and Quality Control

Remaining adapters and poor-quality sequences were trimmed with Trimmomatic (version 0.38) [24] with the following parameters: ILLUMINACLIP:TruSeq3-SE.fa:2:30:10 SLID-INGWINDOW:4:15 MINLEN:15. Sequence quality was then verified using FastQC and summarised with MultiQC (version 1.9) (Figure 1c). Using STAR [25] (version 2.7.5a_202006–19), sequences were aligned to mouse genome assembly m38 with Ensembl version 100 annotations using the quantMode GeneCounts parameter. Mapping quality was verified using MultiQC (Figure 1d,e).

### 3.8. Code Availability

The R code used to analyse and process the raw count data from skin and DRG samples (Table 1) using DESeq2 package version on R version 4.0.3 is publicly available at https://github.com/acirrincio/PaclitaxelRNAseq/ (accessed on 25 May 2022).

## Figures and Tables

**Table 1.** **Raw data descriptor**.

File name	Sample Name	Organism	Strain	Age	Sex	Tissue	Treatment_Timepoint
sRieger_RiegerHarrisonTaxol11202019_201926297-01_S_1_1	SKIN05	Mus musculus	C57B6/J	6 weeks	male	Skin	Vehicle_day4
sRieger_RiegerHarrisonTaxol11202019_201926297-01_S_2_1	SKIN05	Mus musculus	C57B6/J	6 weeks	male	Skin	Vehicle_day4
sRieger_RiegerHarrisonTaxol11202019_201926301-01_S_1_1	SKIN07	Mus musculus	C57B6/J	6 weeks	male	Skin	Vehicle_day4
sRieger_RiegerHarrisonTaxol11202019_201926301-01_S_2_1	SKIN07	Mus musculus	C57B6/J	6 weeks	male	Skin	Vehicle_day4
sRieger_RiegerHarrisonTaxol11202019_201926344-01_S_1_1	SKIN06	Mus musculus	C57B6/J	6 weeks	male	Skin	Vehicle_day4
sRieger_RiegerHarrisonTaxol11202019_201926344-01_S_2_1	SKIN06	Mus musculus	C57B6/J	6 weeks	male	Skin	Vehicle_day4
sRieger_RiegerHarrisonTaxol11202019_201926350-01_S_1_1	SKIN08	Mus musculus	C57B6/J	6 weeks	male	Skin	Vehicle_day4
sRieger_RiegerHarrisonTaxol11202019_201926350-01_S_2_1	SKIN08	Mus musculus	C57B6/J	6 weeks	male	Skin	Vehicle_day4
sRieger_RiegerHarrisonTaxol11202019_201926321-01_S_2_1	SKIN19	Mus musculus	C57B6/J	6 weeks	male	Skin	Vehicle_day7
sRieger_RiegerHarrisonTaxol11202019_201926335-01_S_1_1	SKIN18	Mus musculus	C57B6/J	6 weeks	male	Skin	Vehicle_day7
sRieger_RiegerHarrisonTaxol11202019_201926335-01_S_2_1	SKIN18	Mus musculus	C57B6/J	6 weeks	male	Skin	Vehicle_day7
sRieger_RiegerHarrisonTaxol11202019_201926349-01_S_1_1	SKIN20	Mus musculus	C57B6/J	6 weeks	male	Skin	Vehicle_day7
sRieger_RiegerHarrisonTaxol11202019_201926355-01_S_1_1	SKIN17	Mus musculus	C57B6/J	6 weeks	male	Skin	Vehicle_day7
sRieger_RiegerHarrisonTaxol11202019_201926355-01_S_2_1	SKIN17	Mus musculus	C57B6/J	6 weeks	male	Skin	Vehicle_day7
sRieger_RiegerHarrisonTaxol11202019_201926324-01_S_1_1	SKIN35	Mus musculus	C57B6/J	6 weeks	male	Skin	Vehicle_day11
sRieger_RiegerHarrisonTaxol11202019_201926329-01_S_1_1	SKIN33	Mus musculus	C57B6/J	6 weeks	male	Skin	Vehicle_day11
sRieger_RiegerHarrisonTaxol11202019_201926329-01_S_2_1	SKIN33	Mus musculus	C57B6/J	6 weeks	male	Skin	Vehicle_day11
sRieger_RiegerHarrisonTaxol11202019_201926337-01_S_1_1	SKIN34	Mus musculus	C57B6/J	6 weeks	male	Skin	Vehicle_day11
sRieger_RiegerHarrisonTaxol11202019_201926337-01_S_2_1	SKIN34	Mus musculus	C57B6/J	6 weeks	male	Skin	Vehicle_day11
sRieger_RiegerHarrisonTaxol11202019_201926346-01_S_1_1	SKIN36	Mus musculus	C57B6/J	6 weeks	male	Skin	Vehicle_day11
sRieger_RiegerHarrisonTaxol11202019_201926346-01_S_2_1	SKIN36	Mus musculus	C57B6/J	6 weeks	male	Skin	Vehicle_day11
sRieger_RiegerHarrisonTaxol11202019_201926295-01_S_1_1	SKIN52	Mus musculus	C57B6/J	6 weeks	male	Skin	Vehicle_day23
sRieger_RiegerHarrisonTaxol11202019_201926295-01_S_2_1	SKIN52	Mus musculus	C57B6/J	6 weeks	male	Skin	Vehicle_day23
sRieger_RiegerHarrisonTaxol11202019_201926304-01_S_1_1	SKIN49	Mus musculus	C57B6/J	6 weeks	male	Skin	Vehicle_day23
sRieger_RiegerHarrisonTaxol11202019_201926304-01_S_2_1	SKIN49	Mus musculus	C57B6/J	6 weeks	male	Skin	Vehicle_day23
sRieger_RiegerHarrisonTaxol11202019_201926328-01_S_1_1	SKIN50	Mus musculus	C57B6/J	6 weeks	male	Skin	Vehicle_day23
sRieger_RiegerHarrisonTaxol11202019_201926328-01_S_2_1	SKIN50	Mus musculus	C57B6/J	6 weeks	male	Skin	Vehicle_day23
sRieger_RiegerHarrisonTaxol11202019_201926354-01_S_1_1	SKIN51	Mus musculus	C57B6/J	6 weeks	male	Skin	Vehicle_day23
sRieger_RiegerHarrisonTaxol11202019_201926354-01_S_2_1	SKIN51	Mus musculus	C57B6/J	6 weeks	male	Skin	Vehicle_day23
sRieger_RiegerHarrisonTaxol11202019_201926296-01_S_1_1	SKIN09	Mus musculus	C57B6/J	6 weeks	male	Skin	Paclitaxel_day4
sRieger_RiegerHarrisonTaxol11202019_201926296-01_S_2_1	SKIN09	Mus musculus	C57B6/J	6 weeks	male	Skin	Paclitaxel_day4
sRieger_RiegerHarrisonTaxol11202019_201926306-01_S_1_1	SKIN10	Mus musculus	C57B6/J	6 weeks	male	Skin	Paclitaxel_day4
sRieger_RiegerHarrisonTaxol11202019_201926306-01_S_2_1	SKIN10	Mus musculus	C57B6/J	6 weeks	male	Skin	Paclitaxel_day4
sRieger_RiegerHarrisonTaxol11202019_201926326-01_S_1_1	SKIN12	Mus musculus	C57B6/J	6 weeks	male	Skin	Paclitaxel_day4
sRieger_RiegerHarrisonTaxol11202019_201926339-01_S_1_1	SKIN11	Mus musculus	C57B6/J	6 weeks	male	Skin	Paclitaxel_day4
sRieger_RiegerHarrisonTaxol11202019_201926339-01_S_2_1	SKIN11	Mus musculus	C57B6/J	6 weeks	male	Skin	Paclitaxel_day4
sRieger_RiegerHarrisonTaxol11202019_201926294-01_S_1_1	SKIN25	Mus musculus	C57B6/J	6 weeks	male	Skin	Paclitaxel_day7
sRieger_RiegerHarrisonTaxol11202019_201926294-01_S_2_1	SKIN25	Mus musculus	C57B6/J	6 weeks	male	Skin	Paclitaxel_day7
sRieger_RiegerHarrisonTaxol11202019_201926334-01_S_1_1	SKIN26	Mus musculus	C57B6/J	6 weeks	male	Skin	Paclitaxel_day7
sRieger_RiegerHarrisonTaxol11202019_201926334-01_S_2_1	SKIN26	Mus musculus	C57B6/J	6 weeks	male	Skin	Paclitaxel_day7
sRieger_RiegerHarrisonTaxol11202019_201926336-01_S_1_1	SKIN27	Mus musculus	C57B6/J	6 weeks	male	Skin	Paclitaxel_day7
sRieger_RiegerHarrisonTaxol11202019_201926336-01_S_2_1	SKIN27	Mus musculus	C57B6/J	6 weeks	male	Skin	Paclitaxel_day7
sRieger_RiegerHarrisonTaxol11202019_201926338-01_S_1_1	SKIN28	Mus musculus	C57B6/J	6 weeks	male	Skin	Paclitaxel_day7
sRieger_RiegerHarrisonTaxol11202019_201926338-01_S_2_1	SKIN28	Mus musculus	C57B6/J	6 weeks	male	Skin	Paclitaxel_day7
sRieger_RiegerHarrisonTaxol11202019_201926298-01_S_1_1	SKIN43	Mus musculus	C57B6/J	6 weeks	male	Skin	Paclitaxel_day11
sRieger_RiegerHarrisonTaxol11202019_201926298-01_S_2_1	SKIN43	Mus musculus	C57B6/J	6 weeks	male	Skin	Paclitaxel_day11
sRieger_RiegerHarrisonTaxol11202019_201926310-01_S_1_1	SKIN42	Mus musculus	C57B6/J	6 weeks	male	Skin	Paclitaxel_day11
sRieger_RiegerHarrisonTaxol11202019_201926310-01_S_2_1	SKIN42	Mus musculus	C57B6/J	6 weeks	male	Skin	Paclitaxel_day11
sRieger_RiegerHarrisonTaxol11202019_201926331-01_S_1_1	SKIN44	Mus musculus	C57B6/J	6 weeks	male	Skin	Paclitaxel_day11
sRieger_RiegerHarrisonTaxol11202019_201926331-01_S_2_1	SKIN44	Mus musculus	C57B6/J	6 weeks	male	Skin	Paclitaxel_day11
sRieger_RiegerHarrisonTaxol11202019_201926332-01_S_1_1	SKIN41	Mus musculus	C57B6/J	6 weeks	male	Skin	Paclitaxel_day11
sRieger_RiegerHarrisonTaxol11202019_201926299-01_S_1_1	SKIN60	Mus musculus	C57B6/J	6 weeks	male	Skin	Paclitaxel_day23
sRieger_RiegerHarrisonTaxol11202019_201926299-01_S_2_1	SKIN60	Mus musculus	C57B6/J	6 weeks	male	Skin	Paclitaxel_day23
sRieger_RiegerHarrisonTaxol11202019_201926313-01_S_1_1	SKIN57	Mus musculus	C57B6/J	6 weeks	male	Skin	Paclitaxel_day23
sRieger_RiegerHarrisonTaxol11202019_201926313-01_S_2_1	SKIN57	Mus musculus	C57B6/J	6 weeks	male	Skin	Paclitaxel_day23
sRieger_RiegerHarrisonTaxol11202019_201926319-01_S_2_1	SKIN58	Mus musculus	C57B6/J	6 weeks	male	Skin	Paclitaxel_day23
sRieger_RiegerHarrisonTaxol11202019_201926343-01_S_1_1	SKIN59	Mus musculus	C57B6/J	6 weeks	male	Skin	Paclitaxel_day23
sRieger_RiegerHarrisonTaxol11202019_201926343-01_S_2_1	SKIN59	Mus musculus	C57B6/J	6 weeks	male	Skin	Paclitaxel_day23
							
sRieger_RiegerHarrisonTaxol11202019_201926362-01_S_1_1	DRG08	Mus musculus	C57B6/J	6 weeks	male	DRG	Vehicle_day4
sRieger_RiegerHarrisonTaxol11202019_201926362-01_S_2_1	DRG08	Mus musculus	C57B6/J	6 weeks	male	DRG	Vehicle_day4
sRieger_RiegerHarrisonTaxol11202019_201926366-01_S_1_1	DRG07	Mus musculus	C57B6/J	6 weeks	male	DRG	Vehicle_day4
sRieger_RiegerHarrisonTaxol11202019_201926366-01_S_2_1	DRG07	Mus musculus	C57B6/J	6 weeks	male	DRG	Vehicle_day4
sRieger_RiegerHarrisonTaxol11202019_201926387-01_S_1_1	DRG06	Mus musculus	C57B6/J	6 weeks	male	DRG	Vehicle_day4
sRieger_RiegerHarrisonTaxol11202019_201926387-01_S_2_1	DRG06	Mus musculus	C57B6/J	6 weeks	male	DRG	Vehicle_day4
sRieger_RiegerHarrisonTaxol11202019_201926413-01_S_1_1	DRG05	Mus musculus	C57B6/J	6 weeks	male	DRG	Vehicle_day4
sRieger_RiegerHarrisonTaxol11202019_201926413-01_S_2_1	DRG05	Mus musculus	C57B6/J	6 weeks	male	DRG	Vehicle_day4
sRieger_RiegerHarrisonTaxol11202019_201926361-01_S_1_1	DRG17	Mus musculus	C57B6/J	6 weeks	male	DRG	Vehicle_day7
sRieger_RiegerHarrisonTaxol11202019_201926361-01_S_2_1	DRG17	Mus musculus	C57B6/J	6 weeks	male	DRG	Vehicle_day7
sRieger_RiegerHarrisonTaxol11202019_201926396-01_S_1_1	DRG20	Mus musculus	C57B6/J	6 weeks	male	DRG	Vehicle_day7
sRieger_RiegerHarrisonTaxol11202019_201926396-01_S_2_1	DRG20	Mus musculus	C57B6/J	6 weeks	male	DRG	Vehicle_day7
sRieger_RiegerHarrisonTaxol11202019_201926400-01_S_1_1	DRG19	Mus musculus	C57B6/J	6 weeks	male	DRG	Vehicle_day7
sRieger_RiegerHarrisonTaxol11202019_201926414-01_S_1_1	DRG18	Mus musculus	C57B6/J	6 weeks	male	DRG	Vehicle_day7
sRieger_RiegerHarrisonTaxol11202019_201926414-01_S_2_1	DRG18	Mus musculus	C57B6/J	6 weeks	male	DRG	Vehicle_day7
sRieger_RiegerHarrisonTaxol11202019_201926358-01_S_1_1	DRG34	Mus musculus	C57B6/J	6 weeks	male	DRG	Vehicle_day11
sRieger_RiegerHarrisonTaxol11202019_201926358-01_S_2_1	DRG34	Mus musculus	C57B6/J	6 weeks	male	DRG	Vehicle_day11
sRieger_RiegerHarrisonTaxol11202019_201926364-01_S_1_1	DRG35	Mus musculus	C57B6/J	6 weeks	male	DRG	Vehicle_day11
sRieger_RiegerHarrisonTaxol11202019_201926364-01_S_2_1	DRG35	Mus musculus	C57B6/J	6 weeks	male	DRG	Vehicle_day11
sRieger_RiegerHarrisonTaxol11202019_201926408-01_S_1_1	DRG36	Mus musculus	C57B6/J	6 weeks	male	DRG	Vehicle_day11
sRieger_RiegerHarrisonTaxol11202019_201926408-01_S_2_1	DRG36	Mus musculus	C57B6/J	6 weeks	male	DRG	Vehicle_day11
sRieger_RiegerHarrisonTaxol11202019_201926411-01_S_1_1	DRG33	Mus musculus	C57B6/J	6 weeks	male	DRG	Vehicle_day11
sRieger_RiegerHarrisonTaxol11202019_201926411-01_S_2_1	DRG33	Mus musculus	C57B6/J	6 weeks	male	DRG	Vehicle_day11
sRieger_RiegerHarrisonTaxol11202019_201926370-01_S_1_1	DRG50	Mus musculus	C57B6/J	6 weeks	male	DRG	Vehicle_day23
sRieger_RiegerHarrisonTaxol11202019_201926370-01_S_2_1	DRG50	Mus musculus	C57B6/J	6 weeks	male	DRG	Vehicle_day23
sRieger_RiegerHarrisonTaxol11202019_201926401-01_S_1_1	DRG49	Mus musculus	C57B6/J	6 weeks	male	DRG	Vehicle_day23
sRieger_RiegerHarrisonTaxol11202019_201926401-01_S_2_1	DRG49	Mus musculus	C57B6/J	6 weeks	male	DRG	Vehicle_day23
sRieger_RiegerHarrisonTaxol11202019_201926403-01_S_1_1	DRG52	Mus musculus	C57B6/J	6 weeks	male	DRG	Vehicle_day23
sRieger_RiegerHarrisonTaxol11202019_201926403-01_S_2_1	DRG52	Mus musculus	C57B6/J	6 weeks	male	DRG	Vehicle_day23
sRieger_RiegerHarrisonTaxol11202019_201926405-01_S_1_1	DRG51	Mus musculus	C57B6/J	6 weeks	male	DRG	Vehicle_day23
sRieger_RiegerHarrisonTaxol11202019_201926405-01_S_2_1	DRG51	Mus musculus	C57B6/J	6 weeks	male	DRG	Vehicle_day23
sRieger_RiegerHarrisonTaxol11202019_201926356-01_S_1_1	DRG09	Mus musculus	C57B6/J	6 weeks	male	DRG	Paclitaxel_day4
sRieger_RiegerHarrisonTaxol11202019_201926356-01_S_2_1	DRG09	Mus musculus	C57B6/J	6 weeks	male	DRG	Paclitaxel_day4
sRieger_RiegerHarrisonTaxol11202019_201926399-01_S_1_1	DRG12	Mus musculus	C57B6/J	6 weeks	male	DRG	Paclitaxel_day4
sRieger_RiegerHarrisonTaxol11202019_201926399-01_S_2_1	DRG12	Mus musculus	C57B6/J	6 weeks	male	DRG	Paclitaxel_day4
sRieger_RiegerHarrisonTaxol11202019_201926402-01_S_1_1	DRG10	Mus musculus	C57B6/J	6 weeks	male	DRG	Paclitaxel_day4
sRieger_RiegerHarrisonTaxol11202019_201926402-01_S_2_1	DRG10	Mus musculus	C57B6/J	6 weeks	male	DRG	Paclitaxel_day4
sRieger_RiegerHarrisonTaxol11202019_201926410-01_S_1_1	DRG11	Mus musculus	C57B6/J	6 weeks	male	DRG	Paclitaxel_day4
sRieger_RiegerHarrisonTaxol11202019_201926410-01_S_2_1	DRG11	Mus musculus	C57B6/J	6 weeks	male	DRG	Paclitaxel_day4
sRieger_RiegerHarrisonTaxol11202019_201926360-01_S_1_1	DRG28	Mus musculus	C57B6/J	6 weeks	male	DRG	Paclitaxel_day7
sRieger_RiegerHarrisonTaxol11202019_201926360-01_S_2_1	DRG28	Mus musculus	C57B6/J	6 weeks	male	DRG	Paclitaxel_day7
sRieger_RiegerHarrisonTaxol11202019_201926367-01_S_1_1	DRG27	Mus musculus	C57B6/J	6 weeks	male	DRG	Paclitaxel_day7
sRieger_RiegerHarrisonTaxol11202019_201926367-01_S_2_1	DRG27	Mus musculus	C57B6/J	6 weeks	male	DRG	Paclitaxel_day7
sRieger_RiegerHarrisonTaxol11202019_201926415-01_S_1_1	DRG26	Mus musculus	C57B6/J	6 weeks	male	DRG	Paclitaxel_day7
sRieger_RiegerHarrisonTaxol11202019_201926415-01_S_2_1	DRG26	Mus musculus	C57B6/J	6 weeks	male	DRG	Paclitaxel_day7
sRieger_RiegerHarrisonTaxol11202019_201926417-01_S_1_1	DRG25	Mus musculus	C57B6/J	6 weeks	male	DRG	Paclitaxel_day7
sRieger_RiegerHarrisonTaxol11202019_201926417-01_S_2_1	DRG25	Mus musculus	C57B6/J	6 weeks	male	DRG	Paclitaxel_day7
sRieger_RiegerHarrisonTaxol11202019_201926369-01_S_1_1	DRG43	Mus musculus	C57B6/J	6 weeks	male	DRG	Paclitaxel_day11
sRieger_RiegerHarrisonTaxol11202019_201926369-01_S_2_1	DRG43	Mus musculus	C57B6/J	6 weeks	male	DRG	Paclitaxel_day11
sRieger_RiegerHarrisonTaxol11202019_201926375-01_S_1_1	DRG42	Mus musculus	C57B6/J	6 weeks	male	DRG	Paclitaxel_day11
sRieger_RiegerHarrisonTaxol11202019_201926375-01_S_2_1	DRG42	Mus musculus	C57B6/J	6 weeks	male	DRG	Paclitaxel_day11
sRieger_RiegerHarrisonTaxol11202019_201926382-01_S_1_1	DRG41	Mus musculus	C57B6/J	6 weeks	male	DRG	Paclitaxel_day11
sRieger_RiegerHarrisonTaxol11202019_201926382-01_S_2_1	DRG41	Mus musculus	C57B6/J	6 weeks	male	DRG	Paclitaxel_day11
sRieger_RiegerHarrisonTaxol11202019_201926386-01_S_1_1	DRG44	Mus musculus	C57B6/J	6 weeks	male	DRG	Paclitaxel_day11
sRieger_RiegerHarrisonTaxol11202019_201926365-01_S_1_1	DRG60	Mus musculus	C57B6/J	6 weeks	male	DRG	Paclitaxel_day23
sRieger_RiegerHarrisonTaxol11202019_201926365-01_S_2_1	DRG60	Mus musculus	C57B6/J	6 weeks	male	DRG	Paclitaxel_day23
sRieger_RiegerHarrisonTaxol11202019_201926380-01_S_1_1	DRG59	Mus musculus	C57B6/J	6 weeks	male	DRG	Paclitaxel_day23
sRieger_RiegerHarrisonTaxol11202019_201926380-01_S_2_1	DRG59	Mus musculus	C57B6/J	6 weeks	male	DRG	Paclitaxel_day23
sRieger_RiegerHarrisonTaxol11202019_201926381-01_S_1_1	DRG58	Mus musculus	C57B6/J	6 weeks	male	DRG	Paclitaxel_day23
sRieger_RiegerHarrisonTaxol11202019_201926381-01_S_2_1	DRG58	Mus musculus	C57B6/J	6 weeks	male	DRG	Paclitaxel_day23
sRieger_RiegerHarrisonTaxol11202019_201926393-01_S_1_1	DRG57	Mus musculus	C57B6/J	6 weeks	male	DRG	Paclitaxel_day23
sRieger_RiegerHarrisonTaxol11202019_201926393-01_S_2_1	DRG57	Mus musculus	C57B6/J	6 weeks	male	DRG	Paclitaxel_day23

## Data Availability

All raw and normalised gene count matrices produced by DESeq2 were deposited in the NCBI Gene Expression Omnibus (GEO) database under accession number GSE185084. This GEO entry includes links to the raw count data in .txt.bz2 format. Each processed matrix data file of raw or normalised counts contains a header row corresponding to the sample followed by 52,391 rows, each corresponding to a unique transcript.
